# Dose-Dependent Effects of Multispecies Probiotic Supplementation on the Lipopolysaccharide (LPS) Level and Cardiometabolic Profile in Obese Postmenopausal Women: A 12-Week Randomized Clinical Trial

**DOI:** 10.3390/nu10060773

**Published:** 2018-06-15

**Authors:** Monika Szulińska, Igor Łoniewski, Saskia van Hemert, Magdalena Sobieska, Paweł Bogdański

**Affiliations:** 1 Department of Education and Treatment of Obesity and Metabolic Disorders, University of Medical Sciences in Poznań, Szamarzewskiego Str. 84, 60-569 Poznań, Poland; mszulinska1@wp.pl (M.S.); pawelbogdanski73@gmail.com (P.B.); 2 Department of Biochemistry and Human Nutrition, Pomeranian Medical University in Szczecin, Broniewskiego Str. 24, 71-460 Szczecin, Poland; 3 Winclove Probiotics, Hulstweg 11, 1032 LB Amsterdam, The Netherlands; s.vanhemert@winclove.nl; 4 Department of Rheumatology and Rehabilitation, University of Medical Sciences in Poznań, 28 Czerwca 1956r Str. 135/147, 61-55 Poznań, Poland; msobieska@ump.edu.pl

**Keywords:** probiotics, obesity, metabolic profile, postmenopausal women

## Abstract

During the postmenopausal period, the risk of cardiovascular diseases is increased in many obese women and is associated with a worse cardiometabolic profile and a sub-chronic low-grade systemic inflammation caused by a gut barrier permeability dysfunction. Here, we tested whether administration of two different dosages of the multispecies probiotic Ecologic^®^ Barrier influenced the cardiometabolic biochemical parameters and lipopolysaccharide levels, the latter used as a marker of increased gut permeability in obese postmenopausal women. A total of 81 obese Caucasian postmenopausal women participated in the trial. The subjects were randomly assigned to three groups that received a placebo, a low dose (LD) (2.5 × 10^9^ colony forming units (CFU) per day), or a high dose (HD) (1 × 10^10^ CFU per day) of lyophilisate powder containing live multispecies probiotic bacteria. The probiotic supplement was administered each day in two equal portions for 12 weeks. We found significant (*p* < 0.05) favorable changes (mostly large or medium effects) in the evaluated parameters in both the HD and LD groups but not in the placebo group. In the HD group, lipopolysaccharide, waist, fat mass, subcutaneous fat, uric acid, total cholesterol, triglycerides, low-density lipoprotein cholesterol, glucose, insulin, and insulin-resistant index (HOMA-IR) were improved. Similar changes were observed in the LD group, except for lipopolysaccharide, uric acid, triglycerides, and glucose levels. Additionally, significant differences were observed in both groups in terms of fat percentage and visceral fat. When the mean changes were compared between the three groups, statistically significant differences in lipopolysaccharide levels, uric acid, glucose, insulin, and HOMA-IR were found. Post hoc tests revealed significant differences in the mean changes (mostly medium effects) between the HD and LD groups for uric acid, glucose, insulin, and HOMA-IR. In the 12-week randomized, placebo-controlled, double-blind intervention, we observed that supplementation with the multispecies probiotic Ecologic^®^ Barrier favorably affected the risk factors in a dose-dependent manner, showing beneficial effects on the cardiometabolic parameters and gut permeability of the patients. Our results suggest that this product can be effective in the prevention and treatment of cardiovascular diseases in obese postmenopausal women.

## 1. Introduction

The World Health Organization (WHO) reported that more than 60% of postmenopausal women are overweight or obese [1]. Obesity-associated metabolic disorders, such as dyslipidemia, insulin resistance, hypertension, chronic inflammation, and hyperuricemia, are linked to an increased risk of cardiovascular events [2,3,4], which are the current leading cause of mortality in postmenopausal women [5,6]. Hence, a more focused approach should be adopted for better prevention and treatment of cardiovascular disease (CVD) in obese postmenopausal women [7]. Therefore, new therapeutic interventions are being sought to reduce the cardiometabolic risk in this group of patients. Studies have shown the importance of changes in the intestinal microbiota in the pathogenesis of cardiometabolic disorders [8,9,10,11,12,13]. The intestinal epithelium is a single cell layer that forms a large and important barrier to the external environment. Proper functioning of the intestinal barrier is essential for maintaining optimal health. Increased permeability of the epithelial barrier has been associated with several chronic diseases in humans, including obesity, diabetes, non-alcoholic fatty liver disease, and atherosclerosis [14,15,16,17]. One of the accepted theories that explain the contribution of gut microbes to the development of diseases is sub-chronic inflammation secondary to endotoxemia. This state occurs when fragments of gut-derived Gram-negative bacteria (lipopolysaccharides (LPS) or endotoxin) traverse the intestinal mucosa and enter the circulation, thus facilitating the development of a low-grade systemic inflammation, which is influenced by the host’s metabolic state [17]. Previous studies have also showed that endotoxin can stimulate an innate immune response from the adipose, liver, and skeletal muscle tissues, leading to increased production of proinflammatory cytokines [18]. The human gut microbiome is highly dynamic and can be dramatically altered by antibiotic use and, less rapidly, by age, host genetics, chronic dietary patterns, and other environmental factors [19,20,21,22]. Therefore, the question of whether the administration of probiotics can improve the metabolic parameters in particular groups of patients has arisen. A secondary question concerns the composition and dosage of probiotics that will promote the desired result. A growing body of evidence has suggested that the effects of the probiotics are species- and even strain-specific. Probiotics have been shown to improve the epithelial barrier function in vitro and in vivo in a strain-dependent manner, via different mechanisms [23,24,25]. The strain-specific capacities of the different bacterial strains present in the multispecies probiotic Ecologic^®^ Barrier, a product specially designed to improve the epithelial barrier and to increase its resistance to disturbances, have been recently investigated [26]. The administration of Ecologic^®^ Barrier was shown to improve insulin resistance, evaluated by the homeostasis model assessment for insulin resistance (HOMA-IR), and reduce abdominal adiposity in type 2 diabetes mellitus (T2DM) patients [27]. Although multiple potential effects of probiotics have been studied, no data regarding the influence of the Ecologic^®^ Barrier supplement on the cardiometabolic profile of obese postmenopausal women are available yet.

Therefore, the aim of this study was to evaluate the effect of different doses of the multispecies probiotic Ecologic^®^ Barrier on the LPS level (primary endpoint) and on cardiometabolic parameters (secondary endpoint) in obese postmenopausal women after 12 weeks of supplement administration, by conducting a randomized, placebo-controlled, clinical trial.

## 2. Methods

The study was designed as a 12-week single-center (Department of Education and Treatment of Obesity and Metabolic Disorders University of Medical Sciences in Poznań, Poland), randomized, double-blind, placebo-controlled clinical trial. The protocol was registered at the U.S. National Institute of Health (ClinicalTrails.gov Identifier: NCT03100162). Ethical approval was obtained from the Bioethical Committee of Poznan University of Medical Sciences (No. 871/2015), and written informed consents were obtained from all participants prior to inclusion.

### 2.1. Subjects

**Figure 1:**
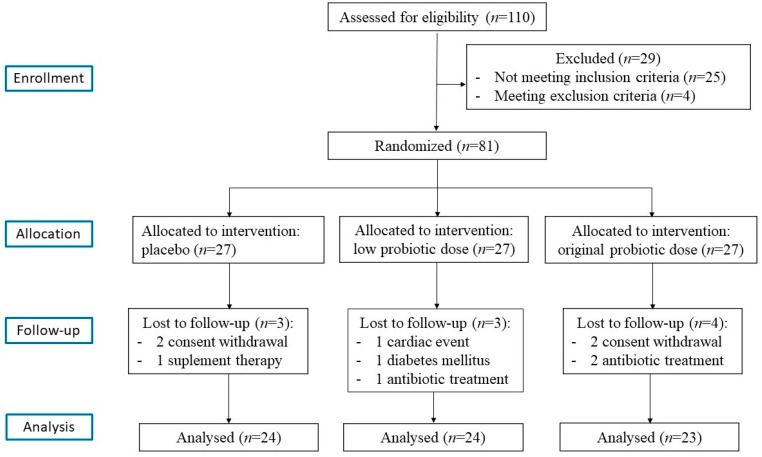
A flowchart of the study design.

All patients were recruited in the outpatient’s department of the University Hospital, Poznań, Poland. A total of 110 obese postmenopausal women were initially invited to participate. The inclusion criteria were as follows: (1) women aged 45–70 years; (2) ≥1 year since last menstruation, (3) body mass index (BMI) 30–45 kg/m^2^; (4) abdominal obesity-related waist circumference >80 cm (International Diabetes Federation 2005); (5) body fat content, assessed by electrical bioimpedance ≥33%; (6) stable body weight in the month prior to the trial (permissible deviation ±1 kg). Patients complying with any of the following exclusion criteria were excluded from the study: (1) secondary form of obesity; (2) gastrointestinal diseases; (3) diabetes; (4) pharmacotherapy for hypertension or dyslipidemia in the 3 months prior to the trial; (5) history of use of any dietary supplements in the 3 months before the study; (6) intake of antibiotics within 1 month before the study; (7) clinically significant acute inflammatory process; (8) nicotine, alcohol, or drug abuse; (9) participation in weight management studies or use of medications known to alter food intake or body weight; (10) vegetarian dietary habits; (11) use of prebiotics- and probiotics-enriched products (for at least 3 weeks before the screening visit) and products with high content of dietary fiber or intake of high quantities of fermented food (>400 g/day); (12) hormone replacement therapy. The compliance to any of the above exclusion criteria during the trial would result in the immediate cessation of participation in the study. On the basis of the inclusion and exclusion criteria, 29 women did not qualify for the study. Finally, 81 women diagnosed with obesity were eligible and provided informed consent. They were randomly assigned to either the placebo or the probiotic group, and this distribution was unknown to both the principal investigators and the participants. Finally, 71 participants (placebo group, *n* = 24, low-dose probiotic (LD) group, *n* = 24, and high-dose probiotic (HD) group, *n* = 23) were able to complete the 12-week intervention. The flowchart of the study is shown in Figure 1.

No serious adverse reactions in the participating postmenopausal women with obesity were reported following the consumption of the multispecies probiotic supplement throughout the study. In addition, the patients did not require any additional medical treatment during the study. The number of patients for whom a follow-up was not possible and the reasons for this are outlined in the flowchart.

### 2.2. Probiotic Supplements and Allocation

All eligible and consenting participants were assigned a unique code as an identifier. They were allocated (1:1:1) to receive either the probiotics (high or low dose) or a placebo. The randomization scheme was computer-generated by Winclove using permuted blocks with block size equal to 4. The research personnel involved in the study was able to adjust the randomization or discern what product the participants were receiving, ensuring the allocation concealment. The probiotic group received sachets containing 2 g of freeze-dried powder of the probiotic mixture Ecologic^®^ Barrier (Winclove probiotics, Amsterdam, The Netherlands). The HD group received Ecologic^®^ Barrier HD (1 × 10^10^ colony forming units (CFU) per day divided in two equal doses), whereas the LD group received Ecologic^®^ Barrier LD (2.5 × 10^9^ colony forming units (CFU) per day divided in two equal doses). The probiotic preparation contained nine bacterial strains: *Bifidobacterium bifidum W23*, *Bifidobacterium lactis W51*, *Bifidobacterium lactis W52*, *Lactobacillus acidophilus W37*, *Lactobacillus brevis W63*, *Lactobacillus casei W56*, *Lactobacillus salivarius W24*, *Lactococcus lactis W19*, and *Lactococcus lactis W58*. All strains are present in approximately equal amounts and quality of the study batch has been tested every 3 months to confirm viability of the strains. This is an updated list compared to that found in previous publications involving Ecologic^®^ Barrier, which results from the application of new molecular identification techniques, including whole-genome sequencing [25]. Since becoming commercially available, this probiotic formulation has always contained the mentioned nine strains and has not been changed in strain content ratios or CFU counts. The placebo group received the same sachets containing only the excipients, i.e., maize starch and maltodextrins. The placebo was indistinguishable in color, smell, and taste from the probiotic formulation. All participants were asked to consume two sachets per day, one before breakfast and one before going to bed, after dissolving the content in a glass of room-temperature water. The participants were asked to return every 4 weeks to hand back the unused sachets and be given fresh refills, so to monitor their compliance to the study protocol. The participants were also asked not to alter their routine physical activity and usual diets and to report any side effects.

### 2.3. Anthropometric and Biochemical Measurement

At enrollment and after 12 weeks of treatment, the anthropometric parameters were evaluated, and all laboratory tests were performed for each group. All measurements were recorded after an overnight fast. The weight was measured to the nearest 0.1 kg, and the height was estimated to the nearest 0.5 cm. The BMI was calculated as weight divided by height squared (kg/m^2^). The waist circumference (cm) was measured between the iliac crest and the lower rib at the end of a normal expiration (to the nearest 0.5 cm). The body fat content (%) was assessed by the electrical bio impedance method using Bioscan 920-2. Fasting serum samples were analyzed for glucose, uric acid, and lipid profile, including total cholesterol (TC), high-density lipoprotein (HDL), and triglycerides (TG), using a Dimension EXL with LM Integrated Chemistry System Analyzer (Siemens, Newark, NJ, USA). The content of low-density lipoprotein cholesterol (LDL) was calculated using the Friedwald equation. Serum insulin (INS) was measured using an immunoradiometric assay (DIAsource immunoassays S.A., Nivelles, Belgium). The sensitivity of the assay, as reported by the manufacturer, has a mean minimum detectable value of 1.0 µIU/mL. Insulin resistance was estimated using the HOMA-IR: HOMA-IR index = (fasting insulin (mU/L) × fasting glucose (mmol/L)/22.5. The serum level of LPS was measured using a quantitative kinetic assay (Lonza, Walkersville, MD, USA). A spike recovery was performed using a sample dilution of 1:40.

### 2.4. Statistical Analysis

The subjects’ randomization codes were concealed until the statistical analysis. The data are shown as mean ± standard deviation (SD). The normal distribution for each group was checked by the Shapiro–Wilk test. To examine the differences among groups, the Kruskal–Wallis test with the post-test (multiple comparison test) or one-way ANOVA test with the post-test (Tukey test) were used if the data were normally distributed. To test the differences between the endpoint and baseline values, the Wilcoxon test or the paired *t*-test were conducted if the data were normally distributed. The standardized mean differences (Cohen’s d) were used as a magnitude of effect. Effect size thresholds of 0.2, 0.5 and 0.8 were used for small, medium and large effects, respectively. Statistics were performed with the STATISTICA data analysis software system, version 12 (StatSoft, Inc., Tulsa, OK, USA, 2014). A *p* value less than 0.05 was regarded as significant.

## 3. Results

The baseline characteristics of the studied population are provided in Table 1 and Table 2. No statistically significant differences were observed in then anthropometric or biochemical parameters among the HD, LD, and placebo groups at baseline (Table 1 and Table 2).

Significant changes in the parameters evaluated before and after the 12 weeks of supplementation were found in both the HD and LD probiotic-supplemented groups but not in the placebo group (Table 3). High-dose probiotic supplementation for 12 weeks decreased LPS by 20.14% (*p* = 0.0008, SMD = −0.77) as well as several other parameters: waist circumference by 1.7% (*p* = 0.0199, SMD = −0.54), fat mass by 3.44% (*p* = 0.03974, SMD = −0.22), subcutaneous fat by 22.91% (*p* = 0.0002, SMD = −0.83), uric acid by 11.13% (*p* = 0.0001, SMD = −0.87), TC by 7.32% (*p* = 0.0019, SMD = −0.57), TG by 7.05% (*p* = 0.014, SMD = −0.43), LDL by 3.99% (*p* = 0.0149, SMD = −0.41), glucose by 7.92% (*p* = 0.0001, SMD = −0.94), INS by 22.41% (*p* = 0.0002, SMD = −0.72), HOMA-IR by 27.27% (*p* = 0.0001, SMD = −0.82); conversely, FFMH was increased by 1.98% (*p* < 0.0129, SMD = 0.39). Low-dose probiotic supplementation did not affect LPS but modified the following parameters: waist circumference by 3.8% (*p* = 0.0001, SMD = −1.06), fat mass by 3.3% (*p* = 0.0099, SMD = −0.62), fat percent by 2.15% (*p* = 0.0103, SMD = −0.54), visceral fat by 11.68% (*p* = 0.0336, SMD = −0.58), subcutaneous fat by 19.00% (*p* = 0.0022, SMD = −0.99), TC by 4.85% (*p* = 0.0124, SMD = −0.49), LDL by 6.36% (*p* = 0.0168, SMD = −0.59), INS by 15.2% (*p* = 0.007, SMD = −0.76), and HOMA-IR by 15. 25% (*p* = 0.0194, SMD = −0.54) (Table 3).

The mean change in the estimated parameters compared among the three groups revealed significant differences in uric acid (*p* = 0.0009), glucose (*p* = 0.0033), INS (*p* = 0.0001), HOMA-IR (*p* = 0.0001), and LPS (*p* < 0.0002). As indicated by the post hoc tests the mean changes differed significantly between HD and placebo (all) as well as between HD and LD (all except for the LPS). The mean change (a reduction) in uric acid (*p* = 0.0109, SMD = −0.73), glucose (*p* = 0.0272, SMD = −0.61), INS (*p* = 0.0002, SMD = −0.83), HOMA-IR (*p* = 0.0005, SMD = −0.90), and LPS (*p* = 0.001, SMD = −0.99) was greater in the HD group when compared to the placebo group. Likewise, the mean change (a reduction) in uric acid (*p* = 0.0016, SMD = −0.92), glucose (*p* = 0.0043, SMD = −0.72), INS (*p* = 0.0155, SMD = −0.40), and HOMA-IR (*p* = 0.0127, SMD = −0.54) was greater in the HD as compared with the LD group (Table 4).

A significant association between the change (Δ) in LPS level (Δ LPS) and Δ HOMA-IR (*r* = 0.40; *p* = 0.04) was found in the LD group, whereas Δ LPS was significantly correlated with Δ waist (*r* = 0.40; *p* = 0.04) in the HD group. No significant relationships were observed in the placebo group (Table 5).

## 4. Discussion

Probiotic supplements have been receiving growing attention due to their potential cardioprotective effects [28,29]. In the present study, we observed that supplementation with the multispecies probiotic Ecologic^®^ Barrier has a beneficial influence on the cardiometabolic profile in obese postmenopausal women.

Our study revealed that, compared with the consumption of a placebo, the consumption of two different doses of multispecies probiotic supplements for 12 weeks reduced waist circumference and visceral and subcutaneous fat in obese postmenopausal patients. Notably, no significant changes were observed in BMI and body weight in the three groups.

Most studies on the anti-obesity effects of probiotics conducted in rodents employed members of the genus *Lactobacillus* [30]. Diet-induced obese mice and diet-induced overweight rats showed a reduction in body weight gain after they were fed specific Lactobacilli [31,32]. Other studies showed that *Lactobacillus gasseri* LG2055 could decrease fat mass and adipocyte size in rodents [33,34]. The anti-obesity effects of probiotics can interfere with the intestinal function. The anorexigenic compounds *N*-acyl-phosphatidylethanolamines (NAPEs) are produced in the small intestine as a response to feeding. Chen et al. genetically modified a probiotic wild-type strain of *Escherichia coli* to secrete NAPEs. The incorporation of bacteria-produced NAPEs in the drinking water of mice fed a high-fat diet for eight weeks resulted in dramatically lower food intake, adiposity, insulin resistance, and hepatosteatosis, compared with the control mice [35].

Promising results in animal models require confirmation in humans. Recently, a systematic review and meta-analysis were published concerning randomized and controlled trials of probiotic supplementation. The effects of probiotics supplementation on body weight, BMI, fat mass, and fat percentage were studied in obese and overweight women and men. The studies included a total of 957 subjects (63% women), with a mean BMI of 27.6 kg/m^2^, and the duration of the interventions ranged from 3 to 12 weeks. The administration of different probiotics resulted in a significant reduction in body weight, BMI, and fat percentage compared with a placebo; however, the effect sizes were small. The effect of the probiotics on fat mass was insignificant [36].

In the studies presented in the meta-analysis, decreases in body weight and BMI were observed. In our study, which seems to be very interesting, the decrease in fat content is independent of body weight and BMI, which did not change significantly. This would testify to the existence of another mechanism that would be responsible for reducing fat percentage content during probiotic therapy in obese women.

Our study is, to the best of our knowledge, the first to report the favorable effect of a multispecies probiotic supplement on the fat mass and other anthropometric parameters in obese post-menopausal women. We believe that the dose and composition of the probiotic used in our study may play an essential role in the achievement of such improvement. The administration of probiotic with the same composition of bacteria (1 × 10^10^ CFU/day for 12 weeks) as was used in our study was tested in a randomized clinical trial conducted by Sabico et al. in medication-naïve T2DM patients [27]. The researchers found that the probiotic supplementation resulted in improved waist/hip ratio and HOMA-IR. Within-group comparisons revealed a significant decrease in the LPS level in the probiotics-receiving group. However, the mean differences in these parameters between the placebo and the probiotics groups were not significant. Within-group comparisons also showed a favorable effect of probiotics intake on glucose, insulin, C-peptide level, and insulin resistance. Probiotics intake also beneficially affected circulating TG and LDL-C. A between-groups analysis did not confirm the significance of the changes in glucose and lipid metabolism. Moreover, no serious side effects were observed. In the probiotic group, four out of 39 patients complained of gastrointestinal discomfort (bloating and flatulence), which disappeared during the first week of treatment.

Our study demonstrates that Ecologic^®^ Barrier and, likely, multispecies probiotics in general, have a beneficial impact on the lipid profile in obese postmenopausal women, as confirmed by the significant decrease in TC and LDL observed after probiotic supplementation in the HD and LD groups and the reduction in TG found in the HD group compared to the placebo group, in which no significant changes were observed. However, the absence of significant differences in other variables among the studied groups shows the need for further research on a larger population. Also, previous in vitro and in vivo studies on probiotics have reported effects on serum lipid profiles. Probiotic administration has been proposed to modulate lipid metabolism in several animal models, including diet-induced obese mice, hypocholesterolemic mice, and hypercholesterolemic rats. Kumar et al. reported the hypocholesterolemic effect of *Lactobacillus plantarum* Lp91 in rats fed a hypercholesterolemic diet and suggested that the indigenous *L. plantarum* strain had the potential to be a successful probiotic in the management of hypercholesterolemia [37]. However, Ejtahed et al. observed that consumption of probiotic yogurt resulted in a nonsignificant decrease in TC and LDL in patients with T2D after six weeks [38]. In another study, the increased serum LDL levels in diabetic patients receiving a probiotic supplement might have resulted from the elevation of serum insulin [39].

Fukushima et al. indicated that a probiotic mixture of organisms including *Bacillus*, *Lactobacillus*, *Streptococcus*, *Clostridium*, *Saccharomyces*, and *Candida* effectively reduced in rats TC and liver cholesterol compared with individual bacteria strains. The effect of the probiotic mixture on Δ6-desaturase activity in liver microsomes was compared with those of *Lactobacillus acidophilus* and *Streptococcus faecalis*. The supplied mixed-bacteria and *L. acidophilus* groups exhibited a 23–57% decrease in cholesterol concentration in the rat liver. Additionally, the serum TC in the supplied mixed-bacteria group was reduced by 15–33% compared with the single-bacteria-supplemented groups [40].

Shimizu et al. conducted a meta-analysis which showed that the administration of probiotics and fermented milk products resulted in changes in TC and LDL concentration greater in mildly hypercholesterolemic than in normocholesterolemic individuals [41]. Along with our results, this meta-analysis data suggest that probiotics can be considered an important element in lipid-lowering therapies.

In this study, we present the effect of a multispecies probiotic supplement on glucose levels in obese postmenopausal women. We observed a significant decrease in glucose, insulin, and HOMA-IR levels in probiotic-supplemented women but not in the placebo group. In addition, these effects were dose-dependent, as confirmed by the significant differences in the mean changes observed between the two supplement-receiving groups and between the treated groups and the placebo group. These results suggest that this probiotic supplement can be effective in the control of glycaemia in obese postmenopausal women.

Specific animal models such as diet-induced obese mice or diabetic mice have been commonly employed to evaluate the effects of probiotics on T2D parameters, to determine the beneficial effects of various strains of Lactobacilli [42,43,44]. Similarly, direct beneficial effects of *Akkermansia mucinifila* on glucose metabolism were identified in a diet-induced T2D mouse model. *A. muciniphila* reduced glucose-6-phosphatase mRNA expression, thus decreasing gluconeogenesis and counteracting fasting hyperglycemia in the diabetic mouse model [45]. The beneficial effects of the consumption of multispecies probiotic supplements on insulin resistance and metabolic profiles, including high-sensitivity C-reactive protein, were also reported in diabetic patients [39]. Sabico et al. showed that supplementation with Ecologic^®^ Barrier significantly improved HOMA-IR and modestly reduced abdominal adiposity in medication-naïve T2DM patients [27]. Probiotic yogurt supplementation controlled the glycemic levels by reducing fasting blood glucose and glycated hemoglobin (HbA1c) in T2D patients. After consuming probiotic yogurt containing *L. acidophilus La5* and *B. lactis Bb12* for six weeks at a dose of 300 g/day, T2D patients experienced a decrease in fasting blood glucose and HbA1c. Additionally, probiotics were found to promote antioxidation in T2D patients [38]. Up to date, most studies concerning the effects of probiotics on the carbohydrate profile have been conducted in animal models, and limited data are available for humans. Some studies have reported beneficial effects of probiotics on serum insulin levels and insulin resistance, but they were restricted to T2D individuals and did not consider obese patients. Moreover, the majority of the previous studies focused on supplementation with monospecies probiotics. Considering the data reported in the literature, our approach consisting in the administration of a multispecies probiotic to obese postmenopausal women is not only innovative but also practically relevant. In fact, our results suggest that a treatment with an available probiotic supplement that can successfully reduce insulin resistance and prevent the development of T2D in this group of patients.

The administration of a multispecies probiotic supplement also significantly decreased serum uric acid level in our study. Hyperuricemia is the basic cause of gout. However, hyperuricemia has also been recognized as a risk factor for arteriosclerosis, cerebrovascular and cardiovascular diseases, and nephropathy. The development of probiotics that efficiently degrade purine compounds is a promising potential therapy for the prevention of hyperuricemia. Li et al. showed the efficacy of the preventive treatment of hyperuricemic rats with probiotic strain DM9218-A. These results suggest that DM9218-A may be a promising candidate for the treatment of patients with hyperuricemia in conjunction with other therapies [46]. DM9218-A may also be effective in the prevention of hyperuricemia in the normal population. Dehghani et al. observed that the intake of a synbiotic supplement, containing both prebiotics and probiotics, could reduce blood urea nitrogen in patients with stages 3 and 4 chronic kidney disease (CKD); however, the other markers of kidney function, including uric acid, were not affected [47]. Asemi et al. investigated the effects of a daily consumption of multispecies probiotic supplements on uric acid level in T2D patients. The researchers did not observe a significant reduction in serum uric acid levels in the treated group compared with the placebo group [39]. Concerning serum uric acid levels, some studies reported increased levels, and others reported decreased levels following probiotic administration [48]. However, the studies on the influence of probiotics on uric acid levels are limited and mainly focused on animal models and on patients with CKD and T2D.

In this study, we demonstrated for the first time that multispecies probiotic supplementation could decrease uric acid levels in obese postmenopausal women in a dose-dependent manner. Elevated levels of circulating insulin were found to increase the intestinal permeability, allowing bacterial toxins, such as LPS, to leak into the circulation, which, in turn, initiated a cascade of inflammatory reactions, thus, explaining the subclinical inflammation present in obese and insulin-resistant patients [49]. Probiotics supplementation can restore the intestinal barrier, preventing LPS influx in the circulation and ultimately reducing subclinical inflammation [50]. By manipulating LPS levels through the introduction of probiotics into the digestive tract, many endotoxin-induced metabolic disorders may, in principle, be reversed.

The results obtained in our study confirm that a probiotic treatment is useful to control LPS levels in obese postmenopausal women. We found that probiotic supplementation not only favorably changed the LPS serum concentration in a dose-dependent manner but also exerted beneficial effects on other parameters, such as waist and HOMA-IR in this population.

## 5. Strengths and Limitations

The strengths of the study are: design (randomized, placebo-controlled, double-blind intervention), wide panel of measured parameters (anthropometric and biochemical), dose-dependent efficacy analysis and test of the marker of gut permeability (LPS).

The major limitation of this study is the relatively small number of individuals examined. The main reason for this was the use of very rigorous inclusion and exclusion criteria. However, the applied criteria enabled us to select a homogenous group of subjects, not affected by diseases or states that might have significantly influenced the results of the study. Another limitation is lack of microbial analysis of feces, which could demonstrate the influence of probiotic bacteria on the gut microbiota composition what can be associated with LPS translocation into the blood circulation. It would be also interesting to perform mechanistic studies (similar to animal models) to explain the favorable effects of metabolic activity of probiotics.

## 6. Conclusions

In this randomized, double-blind, placebo-controlled 12-week trial, we proved for the first time that supplementation with the multispecies probiotic supplement Ecologic^®^ Barrier favorably modified glucose metabolism, lipid profile, waist circumference, visceral fat, serum uric acid level, and LPS concentration in obese postmenopausal women. The role of multispecies probiotic supplements in cardiovascular prevention requires further investigation; however, probiotics supplementation appears to be a useful adjunct therapy for obese patients.

## Figures and Tables

**Table 1:** Baseline characteristics of anthropometric parameters in patients assigned to the high-dose (HD), low-dose (LD), and placebo groups.

Baseline	Group	Mean ± SD	SMD	*p*-Value
Body mass (kg)	High-Dose	94.46 ± 16.61	0.11 *	0.9746
	Low-Dose	92.92 ± 13.66	0.01 †	
	Placebo	92.81 ± 11.93	0.10 #	
BMI (kg/m^2^)	High-Dose	36.57 ± 5.95	0.09 *	0.9365
	Low-Dose	36.00 ± 5.20	−0.02 †	
	Placebo	36.10 ± 4.37	0.10 #	
Age (years)	High-Dose	55.16 ± 6.87	−0.50 *	0.2977
	Low-Dose	56.38 ± 6.55	−0.35 †	
	Placebo	58.72 ± 7.25	−0.18 #	
Height (cm)	High-Dose	160.82 ± 6.23	0.06 *	0.9586
	Low-Dose	160.69 ± 5.43	0.04 †	
	Placebo	160.44 ± 6.38	0.02 #	
Waist circumference (cm)	High-Dose	109.84 ± 11.66	0.09 *	0.9487
	Low-Dose	109.65 ± 10.66	0.08 †	
	Placebo	108.90 ± 7.31	0.02 #	
HR (bpm)	High-Dose	76.26 ± 10.68	0.44 *	0.2466
	Low-Dose	73.58 ± 10.18	0.13 †	
	Placebo	72.48 ± 6.06	0.26 #	
SBP (mmHg)	High-Dose	134.80 ± 10.10	0.10 *	0.7391
	Low-Dose	133.50 ± 10.86	−0.01 †	
	Placebo	133.64 ± 12.20	0.12 #	
DBP (mmHg)	High-Dose	79.88 ± 8.05	−0.51 *	0.1446
	Low-Dose	82.46 ± 5.53	−0.20 †	
	Placebo	83.76 ± 7.26	−0.37 #	
Fat (%)	High-Dose	50.91 ± 6.51	−0.29 *	0.7040
	Low-Dose	51.52 ± 5.34	−0.21 †	
	Placebo	52.85 ± 6.93	−0.10 #	
Fat (kg)	High-Dose	48.48 ± 13.97	−0.02 *	0.9577
	Low-Dose	48.22 ± 11.38	−0.05 †	
	Placebo	48.79 ± 11.02	0.02 #	
FFM (%)	High-Dose	46.88 ± 8.03	0.25 *	0.8152
	Low-Dose	47.06 ± 6.25	0.30 †	
	Placebo	44.91 ± 8.09	−0.03 #	
FFM (kg)	High-Dose	45.95 ± 5.93	0.44 *	0.2934
	Low-Dose	44.70 ± 4.57	0.27 †	
	Placebo	43.03 ± 7.39	0.24 #	
TBW (%)	High-Dose	37.25 ± 5.19	0.26 *	0.8222
	Low-Dose	36.61 ± 4.04	0.16 †	
	Placebo	35.81 ± 5.80	0.14 #	
TBW (I)	High-Dose	35.01 ± 5.11	0.34 *	0.4581
	Low-Dose	33.77 ± 3.56	0.14 †	
	Placebo	33.08 ± 6.22	0.28 #	
FFMH (%)	High-Dose	76.35 ± 3.33	−0.23 *	0.5066
	Low-Dose	75.70 ± 2.50	−0.49 †	
	Placebo	77.07 ± 3.05	0.22 #	
Visceral fat (%)	High-Dose	206.38 ± 66.91	−0.26 *	0.8386
	Low-Dose	218.36 ± 79.02	−0.07 †	
	Placebo	223.77 ± 69.17	−0.16 #	
Subcutaneous fat (%)	High-Dose	297.43 ± 81.90	0.08 *	0.6378
	Low-Dose	278.41 ± 88.98	−0.16 †	
	Placebo	291.27 ± 65.79	0.22 #	

* High-dose vs. placebo; † Low-dose vs. placebo; # High-dose vs. low-dose Data are arithmetic mean ± SD; SMD, standardized mean difference; SBP, systolic blood pressure; DPB, diastolic blood pressure, HR, heart rate; BMI, body mass index; FFM, fat-free mass; TBW, total body water, FFMH, fat-free mass hydration.

**Table 2:** Baseline characteristics of biochemical parameters in patients in the high-dose (HD), low-dose (LD), and placebo groups.

Baseline	Group	Mean ± SD	SMD	*p*-Value
Uric acid (mmol/L)	High-Dose	6.02 ± 0.71	0.50 *	0.0575
	Low-Dose	5.26 ± 1.04	−0.23 †	
	Placebo	5.52 ± 1.23	0.85 #	
TC (mg/dL)	High-Dose	218.56 ± 32.75	0.44 *	0.1377
	Low-Dose	222.27 ± 43.45	0.47 †	
	Placebo	203.60 ± 35.21	−0.10 #	
HDL (mg/dL)	High-Dose	52.48 ± 10.71	0.02 *	0.0912
	Low-Dose	58.27 ± 11.96	0.54 †	
	Placebo	52.32 ± 9.93	−0.51 #	
TG (mg/dL)	High-Dose	165.04 ± 78.15	0.33 *	0.3519
	Low-Dose	134.12 ± 45.98	−0.14 †	
	Placebo	141.76 ± 62.88	0.48 #	
LDL (mg/dL)	High-Dose	119.40 ± 31.86	0.11 *	0.2828
	Low-Dose	129.38 ± 46.81	0.33 †	
	Placebo	115.92 ± 33.47	−0.25 #	
Glucose (mg/dL)	High-Dose	98.60 ± 5.97	0.21 *	0.0620
	Low-Dose	92.81 ± 9.72	−0.29 †	
	Placebo	96.32 ± 14.35	0.72 #	
INS (IU/L)	High-Dose	35.74 ± 12.05	0.59 *	0.0521
	Low-Dose	28.22 ± 10.55	−0.10 †	
	Placebo	29.28 ± 9.87	0.66 #	
HOMA-IR	High-Dose	8.69 ± 3.00	0.65 *	0.0607
	Low-Dose	6.49 ± 2.59	−0.17 †	
	Placebo	6.92 ± 2.46	0.79 #	
LPS (ng/mL)	High-Dose	13.01 ± 5.22	0.54 *	0.0710
	Low-Dose	12.28 ± 6.71	0.33 †	
	Placebo	10.73 ± 3.32	0.12 #	

* High-dose vs. placebo; † Low-dose vs. placebo; # High-dose vs. low-dose Data are arithmetic mean ± SD; SMD, standardized mean difference; TC, total cholesterol; LDL, low-density lipoprotein cholesterol; HDL, high-density lipoprotein cholesterol; TG, triglycerides; INS, insulin; HOMA-IR, homeostasis model assessment for insulin resistant index, LPS, lipopolysaccharide.

**Table 3:** Comparison of the tested parameters in the study groups and the placebo at the beginning of the study and after three months of probiotic (or placebo) supplementation.

Parameter		High-Dose				Low-Dose				Placebo		
	Baseline	After 3 Months	SMD	*p* Value	Baseline	After 3 Months	SMD	*p* Value	Baseline	After 3 Months	SMD	*p* Value
Body mass	94.46 ± 16.61	93.46 ± 14.76	−0.34	0.2173	92.92 ± 13.66	91.82 ± 13.77	−0.39	0.0795	92.81 ± 11.93	92.56 ± 12.37	−0.10	0.5937
BMI	36.57 ± 5.95	36.22 ± 5.29	−0.31	0.3165	36.00 ± 5.20	35.51 ± 5.16	−0.39	0.1209	36.10 ± 4.37	36.04 ± 4.32	−0.07	0.9612
Waist	109.84 ± 11.66	107.97 ± 10.11	−0.54	**0.0199**	109.65 ± 10.66	105.48 ± 11.97	−1.06	**0.0001**	108.9 ± 0.31	107.27 ± 7.16	−0.37	0.0888
Fat%	50.91 ± 6.51	49.54 ± 8.45	−0.41	0.1298	51.52 ± 5.34	50.41 ± 5.60	−0.54	**0.0103**	52.85 ± 6.93	51.38 ± 7.19	−0.40	0.0544
Fat (kg)	48.48 ± 13.97	46.81 ± 14.26	−0.22	**0.0397**	48.22 ± 11.38	46.63 ± 10.53	−0.62	**0.0099**	48.79 ± 11.02	47.75 ± 11.24	−0.29	0.0779
FFM%	46.88 ± 8.03	46.46 ± 10.41	−0.09	0.6649	47.06 ± 6.25	46.66 ± 7.02	−0.16	0.3948	44.91 ± 8.09	45.10 ± 9.35	0.06	0.4118
FMM (kg)	45.95 ± 5.93	45.60 ± 6.82	−0.08	0.7361	44.70 ± 4.57	43.93 ± 4.36	−0.30	0.1870	43.03 ± 7.39	43.19 ± 9.68	0.04	0.4852
TBW%	37.25 ± 5.19	37.83 ± 7.87	0.11	0.6389	36.61 ± 4.04	36.75 ± 4.07	0.08	0.7097	35.81 ± 5.80	36.27 ± 6.62	0.14	0.9095
TBW (Itr)	35.01 ± 5.11	35.24 ± 6.41	0.05	0.5901	33.77 ± 3.56	33.44 ± 3.91	−0.15	0.4928	33.08 ± 6.22	34.10 ± 7.97	0.26	0.4455
FFMH%	76.35 ± 3.33	77.86 ± 3.68	0.39	**0.0129**	75.70 ± 2.50	76.03 ± 3.94	0.19	0.4556	77.07 ± 3.05	77.54 ± 3.39	0.30	0.1492
Visceral fat (%)	206.38 ± 66.91	208.71 ± 66.91	0.03	0.8176	218.36 ± 79.02	192.86 ± 62.38	−0.58	**0.0336**	223.77 ± 69.17	212.14 ± 56.93	−0.23	0.2514
Subcutaneous fat (%)	297.43 ± 81.90	229.29 ± 65.30	−0.83	**0.0002**	278.41 ± 88.98	225.50 ± 59.93	−0.99	**0.0022**	291.27 ± 65.79	241.77 ± 67.28	−0.34	0.0700
Uric acid (mmo/L)	6.02 ± 0.71	5.35 ± 0.91	−0.87	**0.0001**	5.26 ± 1.04	5.28 ± 1.09	0.04	0.8401	5.52 ± 1.23	5.40 ± 1.02	−0.19	0.4004
TC (mg/dL)	218.56 ± 32.75	202.56 ± 30.76	−0.57	**0.0019**	222.27 ± 43.45	211.50 ± 41.39	−0.49	**0.0124**	203.60 ± 35.21	198.08 ± 37.86	−0.18	0.3259
HDL-C (mg/dL)	52.48 ± 10.71	54.68 ± 8.63	0.22	0.1295	58.27 ± 11.96	58.50 ± 11.34	0.02	0.8639	52.32 ± 9.93	55.48 ± 10.76	0.40	0.0511
TG (mg/dL)	165.04 ± 78.15	153.40 ± 55.63	−0.43	**0.0140**	134.12 ± 45.98	123.88 ± 39.51	−0.37	0.0959	141.76 ± 62.88	135.72 ± 69.01	−0.19	0.3002
LDL-C (mg/dL)	119.40 ± 31.86	114.64 ± 37.16	−0.41	**0.0149**	129.38 ± 46.81	121.15 ± 40.62	−0.59	**0.0168**	113.28 ± 35.25	115.92 ± 33.47	0.19	0.3732
Glucose (mg/dL)	98.60 ± 5.97	90.79 ± 8.82	−0.94	**0.0001**	92.81 ± 9.72	92.38 ± 12.29	−0.04	0.8484	96.32 ± 14.35	94.92 ± 8.24	−0.18	0.6373
INS (IU/L)	35.74 ± 12.05	27.73 ± 9.23	−0.72	**0.0002**	28.22 ± 10.55	23.93 ± 8.97	−0.76	**0.0007**	29.28 ± 9.87	29.58 ± 8.39	0.05	0.8119
HOMA-IR	8,69 ± 3.00	6.32 ± 2.47	−0.82	**0.0001**	6.49 ± 2.59	5.50 ± 2.27	−0.54	**0.0194**	6.92 ± 2.46	6.94 ± 2.15	0.01	0.9406
LPS (ng/mL)	13.01 ± 5.22	10.39 ± 5.54	−0.77	**0.0008**	12.28 ± 6.71	11.95 ± 6.84	−0.09	0.2414	10.73 ± 3.32	11.0 ± 3.49	0.17	0.5104

Significant differences (*p* value) are highlighted in bold. The data are arithmetic mean ± SD; SMD, standardized mean difference; BMI, body mass index; Waist–waist circumference, FFM, fat-free mass; TBW, total body water, FFMH, fat-free mass hydration; TC, total cholesterol; LDL-C, low-density lipoprotein cholesterol; HDL-C, high-density lipoprotein cholesterol; TG, triglycerides; INS, insulin; and HOMA-IR, homeostasis model assessment for insulin resistant index.

**Table 4:** Changes in anthropometric and biochemical variables in the high-dose, low-dose, and placebo groups after three months. Significant differences are highlighted in bold.

Variable	Group	Mean ± SD	*p*-Value	SMD	*p*-Value Post-Hoc
Δ Body mass (kg)	High-Dose	−0.99 ± 3.37	0.8611	−0.26 *	ns
	Low-Dose	−1.10 ± 3.07		−0.31 †	
	Placebo	−0.25 ± 2.28		0.03 #	
Δ BMI (kg/m^2^)	High-Dose	−0.35 ± 1.29	0.6960	−0.26 *	ns
	Low-Dose	−0.49 ± 1.29		−0.38 †	
	Placebo	−0.06 ± 0.87		0.11 #	
Δ Waist circumference (cm)	High-Dose	−1.90 ± 3.81	0.1777	−0.06 *	ns
	Low-Dose	−4.17 ± 4.05		−0.58 †	
	Placebo	−1.67 ± 4.27		0.55 #	
Δ Fat (%)	High-Dose	−1.37 ± 4.36	0.9704	−0.68 *	ns
	Low-Dose	−1.10 ± 2.03		−0.84 †	
	Placebo	1.46 ± 3.37		−0.08 #	
Δ Fat (kg)	High-Dose	−1.67 ± 4.49	0.7858	−0.17 *	ns
	Low-Dose	−1.59 ± 2.65		−0.21 †	
	Placebo	−1.03 ± 2.62		−0.02 #	
Δ FFM (%)	High-Dose	−0.42 ± 4.79	0.5319	−0.15 *	ns
	Low-Dose	−0.40 ± 2.37		−0.20 †	
	Placebo	0.19 ± 3.33		−0.01 #	
Δ FMM (kg)	High-Dose	−0.35 ± 4.67	0.9471	−0.11 *	ns
	Low-Dose	−0.76 ± 2.63		−0.25 †	
	Placebo	0.15 ± 4.28		0.11 #	
Δ TBW (%)	High-Dose	0.58 ± 5.72	0.5381	0.03 *	ns
	Low-Dose	0.14 ± 1.78		−0.12 †	
	Placebo	0.46 ± 3.19		0.10 #	
Δ TBW (Itr)	High-Dose	0.23 ± 5.06	0.5598	−0.17 *	ns
	Low-Dose	−0.32 ± 2.17		−0.41 †	
	Placebo	1.02 ± 4.03		0.14 #	
Δ FFMH (%)	High-Dose	1.51 ± 3.52	0.2750	0.38 *	ns
	Low-Dose	0.33 ± 2.06		−0.08 †	
	Placebo	0.47 ± 1.54		0.40 #	
Δ Visceral fat (%)	High-dose	2.33 ± 45.76	0.2281	0.26 *	ns
	Low-Dose	−25.50 ± 52.61		−0.25 †	
	Placebo	−11.64 ± 57.93		0.54 #	
Δ Subcutaneous fat (%)	High-Dose	−68.14 ± 67.54	0.3664	−0.25 *	ns
	Low-Dose	−52.91 ± 71.20		−0.05 †	
	Placebo	−49.50 ± 77.63		−0.22 #	
Δ Uric acid (mmol/L)	High-Dose	−0.68 ± 0.71	**0.0009**	−0.73 *	* 0.0109 # 0.0016
	Low-Dose	−0.02 ± 0.55		0.16 †	
	Placebo	−0.12 ± 0.72		−0.92 #	
Δ TC (mgl/dL)	High-Dose	−16.00 ± 29.24	0.1164	−0.36 *	ns
	Low-Dose	−10.77 ± 22.63		−0.21 †	
	Placebo	−5.52 ± 27.52		−0.20 #	
Δ HDL (mg/dL)	High-Dose	2.20 ± 7.01	0.4023	−0.13 *	ns
	Low-Dose	0.23 ± 13.79		−0.26 †	
	Placebo	3.16 ± 7.70		0.18 #	
Δ TG (mg/dL)	High-Dose	−11.64 ± 39.43	0.7958	−0.16 *	ns
	Low-Dose	−10.23 ± 30.15		−0.14 †	
	Placebo	−6.04 ± 31.46		−0.04 #	
Δ LDL (mg/dL)	High-Dose	−4.76 ± 12.21	0.6503	−0.16 *	ns
	Low-Dose	−8.23 ± 16.37		−0.36 †	
	Placebo	−2.64 ± 14.55		0.24 #	
Δ Glucose (mg/dL)	High-Dose	−7.67 ± 6.88	**0.0033**	−0.61 *	* 0.0272 # 0.0043
	Low-Dose	−0.42 ± 11.17		0.08 †	
	Placebo	−1.40 ± 11.86		−0.72 #	
Δ INS (UI/L)	High-Dose	−8.01 ± 11.30	**0.0001**	−0.83 *	* 0.0002 # 0.0155
	Low-Dose	−4.29 ± 6.40		−0.68 †	
	Placebo	0.30 ± 6.32		−0.40 #	
Δ HOMA-IR	High-Dose	−2.35 ± 2.77	**0.0001**	−0.90 *	* 0.0005 # 0.0127
	Low-Dose	−0.99 ± 2.01		−0.51 †	
	Placebo	0.03 ± 1.86		−0.54 #	
Δ LPS (ng/mL)	High-Dose	−2.62 ± 3.26	**0.0002**	−0.99 *	* 0.001
	Low-Dose	−0.33 ± 3.74		−0.21 †	
	Placebo	0.27 ± 1.60		−0.62 #	

* High-dose vs. placebo; † Low-dose vs. placebo; # High-dose vs. low-dose. Significant differences (*p* value) are highlighted in bold. The data are arithmetic mean ± SD; BMI, body mass index; FFM, fat-free mass; TBW, total body water, FFMH, fat-free mass hydration; TC, total cholesterol; LDL-C, low-density lipoprotein cholesterol; HDL-C, high-density lipoprotein cholesterol; TG, triglycerides; INS, insulin; HOMA-IR, homeostasis model assessment for insulin resistant index, LPS, lipopolysaccharide, SMD, standardized mean difference; ns–not significant.

**Table 5:** The correlation of the change in lipopolysaccharide (LPS) and selected variables in the high-dose (HD), low-dose (LD), and placebo groups after a 12-week intervention.

	Group					
Correlation *n* = 25	HD		LD		Placebo	
	r	*p*-Value	r	*p*-Value	r	*p*-Value
Δ LPS and Δ waist circumference	**0.407**	**0.0436**	−0.229	0.261	0.107	0.6101
Δ LPS and Δ INS	0.272	0.1884	0.306	0.1284	0.220	0.2906
Δ LPS and Δ HOMA-IR	0.301	0.1434	**0.400**	**0.0427**	0.148	0.4811
Δ LPS and Δ LDL	−0.100	0.6346	−0.125	0.5433	0.237	0.2536
Δ LPS and Δ uric acid	0.296	0.1506	−0.189	0.3543	−0.486	0.0738

Significant differences are highlighted in bold. LPS, lipopolysaccharide; INS, insulin; HOMA-IR, homeostasis model assessment for insulin resistant index, LDL, low-density lipoprotein cholesterol.
